# How to Inhibit Nuclear Factor-Kappa B Signaling: Lessons from Poxviruses

**DOI:** 10.3390/pathogens11091061

**Published:** 2022-09-18

**Authors:** Joshua B. Reus, Emily A. Rex, Don B. Gammon

**Affiliations:** Department of Microbiology, University of Texas Southwestern Medical Center, Dallas, TX 75390, USA

**Keywords:** poxvirus, vaccinia virus, NF-κB pathway, immune evasion, virus–host interactions, innate immunity

## Abstract

The Nuclear Factor-kappa B (NF-κB) family of transcription factors regulates key host inflammatory and antiviral gene expression programs, and thus, is often activated during viral infection through the action of pattern-recognition receptors and cytokine–receptor interactions. In turn, many viral pathogens encode strategies to manipulate and/or inhibit NF-κB signaling. This is particularly exemplified by vaccinia virus (VV), the prototypic poxvirus, which encodes at least 18 different inhibitors of NF-κB signaling. While many of these poxviral NF-κB inhibitors are not required for VV replication in cell culture, they virtually all modulate VV virulence in animal models, underscoring the important influence of poxvirus–NF-κB pathway interactions on viral pathogenesis. Here, we review the diversity of mechanisms through which VV-encoded antagonists inhibit initial NF-κB pathway activation and NF-κB signaling intermediates, as well as the activation and function of NF-κB transcription factor complexes.

## 1. Introduction

The *Poxviridae* family comprises a large group of double-stranded (ds) DNA viruses that are unusual among DNA viruses in that they exclusively replicate in the cytoplasm of infected cells. *Poxviridae* can be subdivided into two subfamilies: *Chordopoxvirinae*, which contain members that infect vertebrate hosts, including mammals, birds, and fish [1], and *Entomopoxvirinae,* which include poxviruses that infect insect hosts, such as beetles, grasshoppers, and moths [2]. Within the *Chordopoxvirinae*, the *Orthopoxvirus* genus contains many poxviruses relevant to human health, including variola virus, the causative agent of smallpox, which was one of the deadliest viral diseases in human history [3]. By 1979, the risk of natural smallpox infection had been eliminated as a result of a worldwide vaccination program using attenuated vaccine strains of the closely related orthopoxvirus, vaccinia virus (VV), making smallpox the only human infectious disease ever to be eradicated [4]. Despite the successful eradication of smallpox, other zoonotic poxvirus infections such as monkeypox continue to threaten human health. Indeed, the World Health Organization has recently declared the 2022 outbreak of monkeypox a public health emergency of international concern [5,6]. In addition, poxviruses such as capripoxviruses that cause diseases of veterinary importance are an emerging concern [7]. Importantly, poxviruses may not only be a threat to human health, but might also offer therapeutic strategies for a wide spectrum of diseases. Due to their relatively high recombination rates, the availability of simple recombinant virus construction procedures, the lack of integration into the host genome, and their ability to incorporate >25 kb of foreign DNA, poxviruses are being actively pursued as vectors for gene therapy and for applications in oncolytic virotherapy [8,9,10]. In addition, because poxvirus vectors can stimulate strong humoral and T cell-mediated responses to heterologous antigens, poxviruses are also being applied to the development of vaccines for the treatment of other infectious diseases [11,12,13]. These reasons and the ability of poxviruses to manipulate a wide-range of host processes have made studies of these viruses invaluable in understanding host–pathogen interactions and human disease.

Most of our understanding of poxvirus–host interactions stems from studies with VV, the prototypic poxvirus. During VV infection, a wide range of pattern-recognition receptors such as retinoic acid-inducible gene I (RIG-I)-like receptors (RLRs) and Toll-like receptors (TLRs) can recognize pathogen-encoded molecular patterns (PAMPs) produced by VV, such as dsRNA, to activate innate immune response pathways [10]. Such pathways include the type-I interferon (IFN) response, thought to be the main antiviral defense pathway in mammals, as well as apoptotic and inflammasome-related responses [10]. In addition, VV infection can trigger host antiviral and proinflammatory gene expression through the activation of nuclear factor-kappa B (NF-κB) signaling pathways [11]. Here, we discuss how poxvirus infection can trigger NF-κB activation and how these viruses, in turn, counter these host responses. We focus our discussion on VV because it has been shown to encode at least eighteen individual immune evasion factors that target NF-κB signaling at multiple steps in the pathway, and is an excellent model poxvirus for understanding how this family of viruses can antagonize NF-κB-dependent host responses (Table 1).

## 2. NF-κB Signaling

The innate immune system is the first line of defense against invading microbial pathogens and is comprised of a network of signaling pathways that serve to activate proinflammatory and antimicrobial gene expression programs to combat infection. Many of these signaling pathways converge at NF-κB transcription factor complexes. NF-κB complexes are heterodimeric transcription factors formed from a group of five proteins with N-terminal REL homology domains: NF-κB1/p50, NF-κB2/p52, RelA/p65, RelB, and c-Rel [14]. DNA binding, dimerization, and interaction with inhibitor-κB (IκB) proteins are all mediated by the REL homology domain of NF-κB subunit proteins [15]. The activation of NF-κB can be divided into canonical and non-canonical signaling pathways. The most abundant NF-κB dimer consists of p50/p65 and is involved in the canonical signaling pathway. The p50 and p52 subunits are generated via proteolytic processing of the C-terminal end of their respective precursor proteins, p105 and p100 [16,17]. Both p105 and p100 belong to the IκB family of proteins and play an inhibitory role in NF-κB signaling, but lose their inhibitory function when proteolytically processed during NF-κB signaling activation [18,19]. The activation of the non-canonical NF-κB signaling pathway relies on proteolysis of p100 to p52 and its association with RelB to create active p52/RelB dimers [20]. Non-canonical NF-κB activation is characteristically steady and persistent, while canonical activation is rapid and short-lived. The non-canonical pathway is triggered by ligands of the tumor necrosis factor receptor family and signals through NF-κB inducing kinase (NIK) and IκB kinase alpha (IKKα) [21]. Several viruses have been shown to activate non-canonical NF-κB signaling, which may negatively regulate virus-induced type-I IFN production by competing with active p50/p65 canonical NF-κB complexes for binding to the *Ifnb* locus [22,23,24,25]. In addition to regulating antiviral innate immunity, the non-canonical pathway has also been shown to be involved in functions such as lymphoid development, B cell maturation, and T cell regulation [26,27,28,29,30,31,32,33]. 

In this review, we primarily discuss the canonical pathway because it is the NF-κB pathway most heavily targeted by VV and is the main NF-κB complex contributing to antiviral gene expression. The activation of canonical NF-κB signaling can involve the function of diverse cell-surface and intracellular receptors that respond to a wide range of stimuli [34]. Prior to cellular stimulation, NF-κB remains in the cytoplasm in an inactive state due to the binding of IκB family proteins, which mask NF-κB nuclear localization signals and prevent the complex from entering the nucleus and binding to target genes. The primary upstream receptors that initiate signaling to activate NF-κB are TLRs, tumor necrosis factor alpha receptor (TNFR), and interleukin-1 family receptors (IL-1R) [35]. While TLRs are activated by a variety of PAMPs, such as dsRNA, TNFR and IL-1R signaling is stimulated through binding with pro-inflammatory cytokines, tumor necrosis factor-alpha (TNF-α), and IL-1, respectively. The interaction of these receptors with their ligands (e.g., TNF-α/TNFR and IL-1/IL-1R) triggers a cascade of events involving numerous adaptor proteins and signaling intermediates that ultimately results in NF-κB activation. For example, TNF-α binding to the TNFR on the cell surface triggers the interaction of cytosolic TNFR domains with the homotypic death domain on TNFR-associated death protein (TRADD) and receptor- interacting protein-1 (RIP1) [34]. This interaction facilitates the recruitment of TNF receptor-associated factor-2 (TRAF2) and inhibitor of apoptosis-1/2 (cIAP1/cIAP2), which conjugate K63-polyubiquitin chains to RIP1. The K63-linked ubiquitin chains activate TGF-β-activated kinase-1 (TAK1) as part of the TAK1 kinase complex with TAK-binding proteins 2/3 (TAB2/3) [35]. TAK1 activates the IKK complex (IKKα/IKKβ/IKKγ), resulting in phosphorylation of IκBα by the IKKβ subunit. The E3 ubiquitin ligase SCFβ-transducing repeat-containing protein complex (SCF^β-TRCP^) ubiquitinates IκB in a phosphorylation-dependent manner, targeting it for degradation by the 26S proteasome, and thus, releases NF-κB for nuclear translocation [36,37]. During NF-κB pathway activation through certain TLRs, or through IL-1R or IL-18 receptor (IL-18R) signaling, the adaptor protein myeloid differentiation primary-response gene 88 (MyD88) associates with the toll/interleukin-1 domains (TIR) of these receptors. The death domains of IL-1R-associated kinase-1 and -2 (IRAK1/2) facilitate interaction with MyD88 and initiate a phosphorylation cascade, leading to the activation of TRAF6, which subsequently catalyzes K63-polyubiquitin chain formation and TAK1 activation [38,39]. TAK1 activation results in IKK and IκB phosphorylation, IκB degradation in the proteasome, and subsequent NF-κB activation. 

Additionally, viral DNA and RNA can stimulate cytosolic pattern-recognition receptors such as cyclic GMP-AMP (cGAMP) synthase (cGAS), RLRs, and protein kinase R (PKR) to activate NF-κB signaling. PKR activation triggers an IKK phosphorylation cascade involving phosphorylation at serine 32 and 36 on IκBα, rendering it susceptible to ubiquitination and degradation in the proteasome, thereby releasing active NF-κB for nuclear translocation [40,41,42,43]. Downstream of its initial activation, PKR also phosphorylates eukaryotic initiation factor 2α (eIF2α) at serine 51, rendering the protein inactive, which results in reduced mRNA translation in the cell [44]. While this obstruction in translation is a powerful host defense mechanism to block viral replication, the phosphorylation of eIF2α has also been shown to promote NF-κB-dependent gene expression during stress, as repressed IκBα translation leads to reduced NF-κB inhibition [45]. RLRs recognize dsRNA and initiate NF-κB gene expression by signaling through the mitochondrial antiviral signaling protein (MAVS). MAVS interacts with TRAF6 to activate the IKK complex and phosphorylate IκBα [46]. cGAS binds cytosolic DNA and catalyzes the synthesis of 2′3′-cGAMP from GTP and ATP in a DNA-dependent manner [47]. 2′3′-cGAMP then activates the endoplasmic reticulum-localized stimulator of interferon genes (STING) [48]. Active STING recruits tank-binding kinase 1 (TBK1), which functions in parallel to phosphorylate interferon regulatory factor 3 (IRF3) and activate the IKK complex [49]. Phospho-IRF3 forms a dimer and functions as a transcription factor for type-I interferons, while the IKK complex phosphorylates IκBα to activate NF-κB.

Besides regulation by IκB, NF-κB activity can also be controlled by direct post-translational modifications of NF-κB subunits. For example, site-specific acetylation of p65/RelA can regulate the transcriptional activity of NF-κB. Three known sites on p65/RelA, lysine 218, 221, and 310 are acetylated by CREB-binding protein (CBP) and p300, which, in turn, promote NF-κB transcriptional activity [50]. In contrast, deacetylation by histone deacetylases (HDAC), such as HDAC3, enhance binding to IκBα, inactivate NF-κB, and promotes its nuclear export [51].

NF-κB regulates a considerable number of genes involved in immunity, inflammation, cell growth and development, and apoptosis [52,53,54,55]. One of the most extensively studied antiviral factors that is regulated by NF-κB is interferon-β (IFN-β). IFN-β plays a key role in arming cells by upregulating antiviral factors that restrict infection known as interferon-stimulated genes (ISGs) [56] (note: the role of the IFN pathway during poxvirus infection has been recently reviewed elsewhere [10,57,58,59]). NF-κB maintains basal levels of IFN-β in normal cells and rapidly induces IFN-β after viral infection [60,61,62]. In addition, there are several other well-characterized antiviral factors transcriptionally up-regulated by NF-κB such as: ISG15, IFIT1, CCL5/RANTES, and GBP2 [53,63,64,65,66]. Gene targets of NF-κB encoding transcription factors such as IRF7 can, in turn, induce additional subsets of ISGs [67]. This highlights the importance of the NF-κB signaling pathway in inducing antiviral immunity and explains why VV has evolved diverse strategies to counteract this pathway.

## 3. VV Inhibitors Targeting Receptors Mediating NF-κB Activation

### 3.1. E3

**Figure 1:**
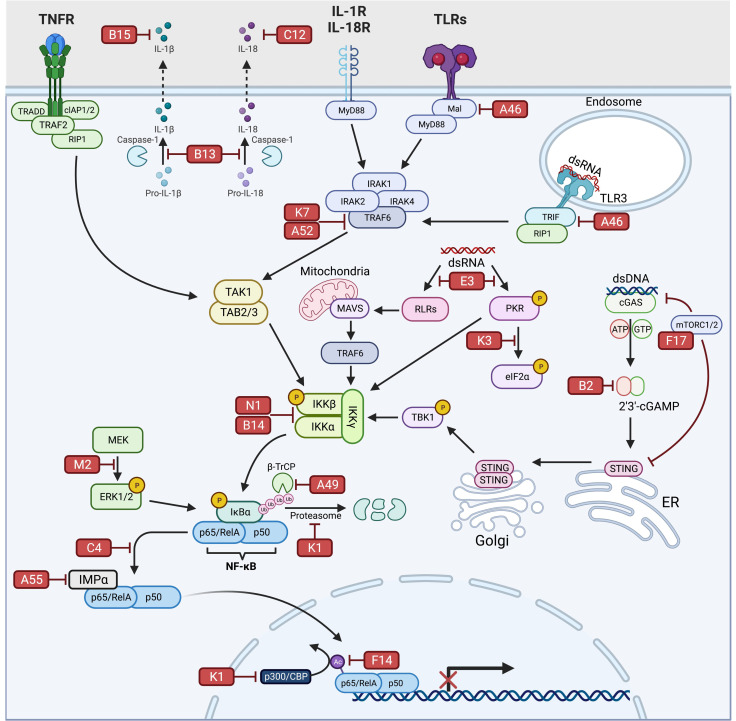
Inhibition of NF-κB signaling by VV. An overview of the various stages of antiviral NF-κB signaling targeted by VV. The 18 known inhibitors are shown as red rectangles positioned next to the component they are thought to inhibit. See text for additional details. TNFR: tumor necrosis factor-alpha receptor; IL-1R: interleukin-1 receptor; IL-18R: interleukin-18 receptor; TLRs: Toll-like receptors; MyD88: myeloid differentiation primary response gene 88; Mal: MyD88-adapter-like; TRADD: Tumor necrosis factor receptor type-1-associated death domain protein; cIAP1/2: cellular inhibitor of apoptosis 1/2; RIP1: receptor-interacting protein 1; TRAF2/6: tumor necrosis factor receptor-associated factor 2/6; IL-1β: interleukin-1β; IL-18: interleukin-18; IRAK1/2/4: interleukin-1 receptor-associated kinase 1/2/4; TRIF: Toll/interleukin-1 receptor domain-containing adapter-inducing interferon-β; TAK1: Transforming growth factor-β (TGF-β)-activated kinase 1; TAB2/3: TGF-β-activated kinase 1-binding protein 2/3; RLRs: RIG-I-like receptors; MAVS: mitochondrial antiviral-signaling protein; cGAS: cyclic AMP-GMP synthase; cGAMP: cyclic AMP-GMP; STING: stimulator of interferon genes; TBK1: TANK-binding kinase 1; mTORC1/2: mammalian target of rapamycin; PKR: protein kinase R; dsRNA: double-stranded RNA; eIF2α: eukaryotic translation initiation factor; IκBα: inhibitor κBα; IKKα: IκBα kinase α; IKKβ: IκBα kinase β; p65/p50: NF-κB heterodimer p50/p65 subunit; CBP: CREB-binding protein; MEK: MAPK/ERK kinase; ERK1/2: extracellular signal-regulated kinase 1/2; IMPα: importin-α; β-TrCP: β-transducin repeat-containing E3 ubiquitin protein ligase; NF-kB: Nuclear Factor-kappa B. Figure was created using Biorender.com, accessed on 1 August 2022.

One of the mechanisms by which VV inhibits upstream activation of the NF-κB pathway is by actively antagonizing PKR activity [68,69] (Figure 1). Despite being a DNA virus, bidirectional transcription of VV mRNA from both genomic DNA strands produces overlapping transcripts that can form dsRNA and activate PKR signaling [70]. One of the best-characterized VV-encoded inhibitors of PKR is E3, encoded by the *E3L* gene. E3 is non-essential for replication in certain cell types and localizes to both the cytoplasm and nucleus under infection or overexpression conditions [71]. E3 uses its C-terminal dsRNA-binding domain to sequester viral dsRNA products during infection to prevent PKR activation [72,73,74]. The dsRNA-binding domain of E3 shares sequence similarity with not only other mammalian poxvirus-encoded E3 orthologs, but also with known cellular dsRNA-binding domain-containing proteins, such as RNase II and PKR itself [75]. Interestingly, the N-terminal domain has been reported to directly interact with the protein kinase domain of PKR in in vitro pulldown assays [76] and functions to inhibit PKR by forming non-functional heterodimers [73]. Thus, both the N- and C-terminal domains can antagonize PKR through independent mechanisms. In human HAP1 cell cultures, Δ*E3L* VV strains fail to replicate; however, genetic inactivation of PKR is sufficient to relieve this restriction phenotype, indicating the importance of E3-mediated PKR antagonism in promoting VV replication [77]. In addition to PKR antagonism, E3 dsRNA-binding activity has been shown to be required for the inhibition of RLR-mediated NF-κB gene expression through MAVS [78]. During VV infection, cytosolic dsRNA recognition by cellular RLRs is inhibited by the sequestration of this dsRNA by E3, leading to a block in downstream NF-κB activation [78].

The importance of both E3 domain immune evasion functions to pathogenesis was revealed through intranasal mouse model studies comparing WT to strains encoding either full Δ*E3L* deletions or N- or C-terminal domain deletions. While WT VV displayed a lethal dose 50 (LD_50_) of 10^3^–10^4^ plaque-forming units (PFU), strains encoding full deletions or N- or C-terminal deletions all exhibited LD_50_ values greater than 10^7^ PFU [79], suggesting that both domains are necessary for virulence [80].

### 3.2. K3

In addition to blocking PKR activation by dsRNA, VV also encodes K3, the product of the early *K3L* gene, which associates with PKR and serves as a non-phosphorylatable pseudo-substrate of PKR that impedes the phosphorylation of PKR targets such as eIF2α [81,82,83]. First identified as an eIF2α mimic, K3 structurally resembles the eIF2α N-terminal domain [84] and in vitro competition assays suggest that PKR recognizes both eIF2α and K3 via similar mechanisms [76]. However, despite K3′s characterized involvement in PKR inhibition, *K3L* deletion has little effect on VV replication in cell culture or PKR-induced responses in various human cell types [85,86,87]. Intratracheal models of infection in mice suggest that K3 does not exclusively target PKR in vivo, as Δ*K3L* infections showed no virulence differences in PKR^−/−^ mice; however, this study found that VV Δ*K3L* strains failed to disseminate from the lung to other tissues, indicating a possible function in facilitating virus spread [88]. Thus, it appears that E3 accounts for the majority of anti-PKR activity, inhibiting NF-κB induction during VV infection. 

### 3.3. K1

The early gene product of *K1L*, K1, was previously shown to inhibit IκBα degradation in rabbit RK13 cells [89]. Compared to the Western Reserve (WR) VV strain, the attenuated Modified Vaccinia Ankara (MVA) strain lacks many immunomodulatory genes, including *K1L*, and induces a robust NF-κB response in mammalian cells [89]. Genetic complementation of the functional WR *K1L* gene into the MVA strain demonstrated that K1 is sufficient for inhibiting the degradation of IκBα, thereby repressing the transcription of NF-κB-regulated genes [89]. Structural analyses identified nine ankyrin repeats encoded within K1. Ankyrin repeats are a ~33-residue eukaryotic motif that mediate protein–protein interactions [90] and are rarely observed in viral proteins with the exception of the *poxviridae* family. However, the role of these ankyrin repeats in K1-mediated NF-κB inhibition is still unknown [91]. Interestingly, an increase in dsRNA levels from early viral gene transcription has been reported in Δ*K1L* strain infections that triggers PKR activation, and only infections with functional K1 present can prevent subsequent NF-κB activation, suggesting that K1 additionally plays a role in reducing dsRNA levels early during infection to minimize PKR stimulation and downstream NF-κB activation [70]. These observations suggest a critical role for K1 in suppressing PKR-mediated NF-κB activation even in the presence of E3 and K3. In vivo, VV strains lacking *K1L* exhibit decreased virulence when inoculated via either intranasal or intradermal routes, further confirming a key role for K1 in modulating VV pathogenesis [92].

### 3.4. C12

The non-essential, early VV gene product of *C12L* was initially hypothesized to be a soluble IL-18-binding protein (IL-18BP) due to its high sequence similarity to other human and mouse IL-18BPs [93]. IL-18BPs are negative regulators of the IL-18 pro-inflammatory cytokine, suggesting that C12 may similarly function to antagonize IL-18 signaling [94]. Indeed, upon VV infection, C12, also known as vIL-18BP, is secreted outside of the cell and binds specifically to IL-18 in solution, preventing IL-18 interaction with IL-18R [93,95]. Though the VV C12 structure remains unsolved, the ectromelia (ECTV) homolog, ectvIL-18BP, was crystallized to a 2.0-Å resolution in a complex with human IL-18 [96]. The 95% sequence similarity between C12 in VV and ECTV provides valuable functional insight to the interaction interface concerning VV C12 and human IL-18 [96]. ectvIL-18BP stoichiometrically interacts with IL-18 by inducing a conformational change, allowing ectvIL-18BP to clamp to the binding interface of the IL-18 β-barrel [96]. ectvIL-18BP therefore neutralizes IL-18 and prevents further interaction with IL-18R. The inhibition of IL-18R activation precludes downstream MyD88- and TRAF6-mediated NF-κB signaling (Figure 1) [94]. Intranasal models of VV infection in mice suggests that C12 is a key virulence factor as mice experienced minimal weight loss after Δ*C12L* strain inoculation, and viral loads recovered from the brain, lungs, and spleen were significantly reduced compared to WT strain infections [97]. In addition, intracranial models demonstrated a ~10 times higher LD_50_ in Δ*C12L* strain infections compared to inoculations with parental strains, underscoring the contribution of C12 function to VV pathogenesis [98].

### 3.5. B15

B15, or vIL-1βR, is the soluble, secreted product of *B15R*, a non-essential gene expressed early during infection [99]. It has been characterized as an IL-1-binding protein due to significant sequence similarity with IL-1R [100]. IL-1R utilizes the MyD88/TRAF6 pathway upon receptor stimulation to activate NF-κB responses, triggering the transcription of pro-IL-1β genes. Pro-IL-1β matures upon caspase-1 cleavage into intracellular IL-1β, then is secreted, consequently amplifying the IL-1β cytokine response (Figure 1) [101]. Ligand blots were one of the first methods used to experimentally demonstrate that B15, concentrated from VV infected supernatants, can bind to IL-1 [102]. IL-1R binding inhibition assays showed that concentrated supernatants from WT, but not Δ*B15R*, VV infections prevent IL-1β from binding to IL-1R. B15 has s strong affinity for IL-1β, with a K_D_ of 234 pM which is comparable to the affinity of cellular IL-1 receptors [103]. Interestingly, B15 contributes to VV virulence in a manner dependent upon the route of infection. For example, Δ*B15R* WR strains exhibited a 285-fold increase in LD_50_ compared to parental strains in intracranial infection models in mice [102]. However, in intranasal models of infection, no significant differences in mortality were observed between WT and Δ*B15R* strains [103]. Still, infection-related symptoms (e.g., lessened mobility, arched backs, and ruffled fur) appeared earlier and were exacerbated in the Δ*B15R* strain infections, suggesting that IL-1R signaling promotes pathogenic inflammatory responses during infection, and that B15 functions to dampen these responses [103].

## 4. VV Inhibitors Targeting NF-κB Signaling Intermediates

### 4.1. K7

*K7R* is an early, non-essential gene encoding the K7 NF-κB pathway antagonist [104]. Like several other VV antagonists targeting the NF-κB pathway (discussed below), the K7 protein adopts a fold characterized as a B-cell lymphoma (Bcl)-2-like structure and was one of the initial members to be identified in this Bcl-2-like subgroup [105]. Notably, K7 has binding affinities for multiple NF-κB pathway signaling intermediates including TRAF and IRAK2 [104]. Through these interactions, K7 has been shown to antagonize TLR-activated NF-κB signaling [106]. Co-immunoprecipitation assays suggest that K7 interaction is mediated through the TRAF domain (a.a. 289–522) on TRAF6 [106]. TRAF6 is also essential for IL-1α-induced NF-κB activation, suggesting that K7 inhibition can prevent IL-1α-stimulated NF-κB induction. In vivo, Δ*K7R* VV strains were attenuated in both intradermal and intranasal mouse models [104]. Intradermal infections with Δ*K7R* strains induced smaller lesions, and intranasal infections led to more rapid clearing of the infection in lung tissues compared to the parental strain [104]. Moreover, upon examination of the intrapulmonary innate immune response, elevated macrophage-dependent antigen presentation, immune cell infiltration, and cytolysis of infected cells by natural killer and CD8+ T-cells was observed during Δ*K7R* intradermal infections [104]. Together, this suggests that K7 is a bona fide virulence factor regardless of the route of VV infection.

### 4.2. A46

A46 is encoded by the early gene, *A46R*, and localizes near the cytosolic face of the plasma membrane [107,108]. A46 was one of the first reported viral proteins to encode a domain with similarity to cellular Toll/IL-1 (TIR) domains found in the IL-1/TLR superfamily of receptors [109]. A46 interacts with a diverse set of host TIR domain-containing adaptor proteins such as MyD88, as well as the upstream factor Myd88-adaptor-like (Mal), thereby impeding their promotion of TLR-mediated NF-κB signaling [107] (Figure 1). Mal is required for recruiting MyD88 to TLRs, while MyD88 influences subsequent downstream signaling to NF-κB, not only as a result of TLR activation but also after IL-1R or IL-18R activation [110,111]. Consequently, A46 can suppress multiple PAMP- and cytokine-initiated pathways for activating NF-κB transcription by interfering with common signaling intermediates such as MyD88 [112]. The crystal structure of A46 revealed a Bcl-2-like fold and a TLR-inhibitory motif involved in Mal binding [113]. Further structural studies suggest that the A46 C-terminal region interacts with Mal, while MyD88 interaction is limited to the N-terminus, and that A46 could simultaneously interfere with multiple other TIR-domain-containing host proteins (e.g., TRIF and TRAM) involved in NF-κB activation [114]. Intranasal mouse models of WT and Δ*A46R* VV infections revealed that strains lacking A46 displayed reduced virulence compared to WT strains [107], suggesting that the obstruction of multiple TIR domain containing proteins contributes significantly to poxvirus pathogenesis. 

### 4.3. A52

*A52R* encodes an additional early gene product, A52, with cytoplasmic localization, which also disrupts TLRs and cytokine-dependent NF-κB signaling [109,115]. Like A46, A52 also adopts a Bcl-2-like fold and was identified as a putative antagonist of host TIR domain-containing proteins based on the identification of a TIR domain within A52 [109,116]. Through overexpression studies, A52 was shown to effectively inhibit MyD88-dependent NF-κB activation through IL-1R, IL-18R, and TLRs [109]. This suggested that A52 was acting on a common signaling intermediate downstream of these receptors, which is critical for NF-κB activation. Co-immunoprecipitation assays subsequently revealed that A52 complexes with IRAK2 and TRAF6 signaling intermediates [117]. A52-IRAK2 interaction is mediated though the death domain on IRAK2, and as A52 expression increases, IRAK2 complex formation with Mal decreases, suggesting a mechanism for how A52 prevents downstream NF-κB induction [117]. Similarly, A52 obstructs TRAF6 from binding to its downstream signaling partner, TAB1, preventing NF-κB induction by inhibiting TRAF6-TAB1 complex formation [117]. A52 interacts with TRAF6 through the TRAF6-encoded TRAF domain and has been shown to enhance virulence in murine intranasal models of infection [117].

### 4.4. B14

*B14R* is a non-essential, early gene that encodes the diffusely cytosolic B14 protein [118]. Despite Δ*B14R* strains having no significant differences in pathogenesis in an intranasal murine model, studies with intradermal models of infection found Δ*B14R* strains to produce smaller lesions compared to parental strains [118]. Titers collected from infected lesions were also reduced in Δ*B14R* strains [118]. Similar phenotypes are observed in cell culture, where Δ*B14R* mutant plaques are smaller compared to parental WR and revertant strains [118]. 

Early bioinformatic analyses suggested that B14 belonged to the growing family of orthopoxvirus proteins found to encode a Bcl-2-like fold (e.g., A46, A52, N1, and K7) and that were also known for inhibiting pathways leading to IFN and/or NF-κB activation [119,120]. Ectopic expression of B14 was subsequently shown to inhibit TNF-α- and IL-1β- induced NF-κB activation [121]. Compared to WT Infections, Δ*B14R* strain-infected cells exhibited increased IκBα phosphorylation, suggesting that B14 may target IKK, upstream of IκBα [121]. Subsequent work showed that purified B14 protein co-precipitated with both human and mouse IKK complexes through interaction with the IKKβ subunit [121]. The mapping of interaction sites revealed that B14 docks at phosphorylation sites present at residues S177 and S181 in the IKKβ activation loop [121]. Structural analyses suggest that B14 interaction prevents IKKβ trans-auto-phosphorylation and activation; additionally, it sterically hinders IKKβ-IKK complex formation, dampening total IKK activity during infection [122]. This reduced IKK activity ultimately impedes phosphorylation and the proteasome-mediated degradation of IκBα, maintaining NF-κB in an inactive state. 

### 4.5. N1

Like many other VV immunomodulators, *N1L* is expressed early during infection, and its protein product, N1, localizes to the cytoplasm. Like B14 and A52, N1 also adopts a Bcl-2-like fold [123,124]. Unlike other NF-κB antagonists, however, N1 also inhibits apoptosis (note: see Veyer et al. for a more extensive discussion of N1-mediated regulation of apoptosis [125]). 

N1 overexpression in HEK293 cells significantly suppressed NF-κB stimulation through both IL-1β and TNF-α signaling pathways [126,127]. This is in contrast to A52, which does not block TNF-α-stimulated NF-κB activation, suggesting that N1, despite sharing sequence similarity with A52, exhibits a functionally distinct mechanism for antagonizing NF-κB [127]. N1 has been reported to inhibit NF-κB signaling by interacting with members of the IKK complex that facilitate NF-κB activation [127]. However, it should be noted that other studies have failed to identify the interaction between N1 and components of the IKK complex [121,123]. This disparity may, in part, be due to the subtle influence N1 may have during infection in the presence of other VV NF-κB inhibitors. Regardless, the precise mechanism of N1 remains unresolved. Interestingly, mutagenesis studies identified an N1 substitution mutant, I6E, which prevents N1 protein homodimerization and N1-mediated NF-κB inhibition [126]. Although the way in which dimer formation contributes to NF-κB pathway inhibition is still unclear, intranasal inoculation of mice using either the Δ*N1L* strain or strains encoding the I6E N1 mutant were typified by reduced virulence compared to WR infections, suggesting that N1 dimerization and antagonism of NF-κB contributes to VV pathogenesis [126].

### 4.6. B13

*B13R* encodes B13, also known as serine-protease inhibitor (SPI)-2, and is a non-essential protein expressed early during VV infection [128,129]. Through sequence analysis, B13 was first speculated to function as a serpin, a family of serine protease inhibitors, due to its similarity to the cowpox virus-encoded cytokine response modifier (CrmA) [130]. Despite near-perfect sequence conservation between WR and its CrmA counterpart in cowpox, not all VV strains encode functional B13. For example, in the Copenhagen strain, B13 is fragmented due to a downstream frame-shift mutation caused by an N-terminal truncation [119,129,131]. In MVA, *B13R* is also non-functional, as the gene is fragmented [132,133]. 

B13 functions as a pan-caspase inhibitor, preventing the caspase-mediated cleavage of apoptotic and NF-κB-related factors [134]. Examples of the latter include pro-IL-1β and pro-IL-18β; both are NF-κB-induced gene products that are cleaved by caspase-1, producing mature IL-1β or IL-18β, respectively; this can, in turn, activate NF-κB signaling (Figure 1) [135]. B13 blocks IL-1β maturation by preventing caspase-1 cleavage, as Western blot and caspase-1 inhibition assays did not detect mature IL-1β products in the presence of B13, despite the presence of the pro-IL-1β precursor in human THP-1 cells [128]. Pro-IL-18β is also regulated by the same caspase in a similar manner [136]. Thus, caspase-1 inhibition leads to decreased IL-1 and IL-18 cytokine abundance, reducing NF-κB pathway amplification via autocrine and paracrine signaling [137]. 

The mechanism of B13 caspase-1 inhibition in VV has yet to be determined. However, the related cowpox CrmA has been shown to tightly interact with caspase-1 in vitro through a C-terminal reactive-site loop on CrmA [138]. This interaction was later corroborated by structural studies demonstrating that CrmA docked onto caspase-1 via the C-terminal loop [139]. This direct interaction between CrmA and, presumably, B13 is thought to prevent the caspase-1-mediated cleavage of IL-1β.

Interestingly, Δ*B13R* deletion in the WR strain did not affect virulence in intranasal mouse models [129], though intradermal infections with this mutant led to larger lesions compared to the parental WR strain, suggesting that B13 functions to limit host immune response-related pathology [140]. 

### 4.7. B2

*B2R* encodes B2, also referred to as “poxin”, which stands for poxvirus-induced nuclease [141]. Expressed early during infection, B2 is a specific 2′3′-cGAMP-degrading enzyme [115,141]. Biochemically, B2 linearizes 2′3′-cGAMP at the 3′-5′ bond, leaving behind a Gp[2′–5′]Ap[3′] product [141]. Electrophoretic mobility shift assays demonstrated that STING no longer recognizes the cleaved form of 2′3′-cGAMP, and thus, B2 impedes STING from activating downstream immune responses [141]. Radiolabeling of cGAMP in African green monkey cells demonstrated that 2′3′-cGAMP degradation occurs within the first hour of infection with the WR strain of VV [141]. In contrast, the Δ*B2R* strain failed to degrade 2′3′-cGAMP during infection [141]. In skin-scarification-infection-model studies in mice, the Δ*B2R* virus replicated to levels ~40 times lower than the parental WR strain, suggesting that the cGAS/STING pathway significantly contributes to poxvirus restriction during infection of the skin [141].

### 4.8. F17

Until recently, the gene product of F17R, formerly F18, was classically characterized as an essential, highly abundant structural protein expressed late during VV infections, with no known non-structural function described [115,142,143]. However, F17 was later identified as a dysregulator of mammalian target of rapamycin complexes (mTORC1/2) via the sequestration of mTORC1/2 master regulators, Raptor and Rictor. The sequestration of Raptor and Rictor by F17 leads to the hyperactivation of mTOR and subsequent cGAS degradation, thereby blocking downstream STING-mediated NF-κB signaling [144].

## 5. VV Inhibitors Directly Targeting NF-κB Complex Activation/Activity

### 5.1. A49

The *A49R* gene encoding the A49 protein is transcribed both early and late during VV infection [115]. Studies in which *A49R* was deleted showed that A49 is non-essential and does not significantly impact viral replication in cell culture [145]. During NF-κB pathway activation, the E3 ligase, β-transducin repeat-containing protein (β-TrCP) ubiquitinates phosphorylated IκBα (p-IκBα), which leads to the proteasomal degradation of p-IκBα [37], permitting the nuclear translocation of active NF-κB (Figure 1). A49 mimics the “SXXXS” binding motif found in IκBα and other substrates of β-TrCP and prevents p-IκBα degradation via β-TrCP ubiquitination [145]. Mansur et al. showed that A49-expressing VV strains promote the accumulation of cellular p-IκBα levels in the presence or absence of TNF-α stimulation, while Δ*A49R* knockout strains do not [145]. These observations suggest that A49 can inhibit NF-κB activation stimulated by viral infection or cytokine signaling, and thus, can act as a potent inhibitor of NF-κB.

The β-TrCP-binding motif of A49, “SGNLES”, encodes a serine residue at position 7 that requires phosphorylation by IKKβ in order to bind to β-TrCP and inhibit NF- κB [146]. VV A49 mutants encoding an alanine in place of this serine residue display reduced virulence that is intermediate between the Δ*A49R* knockout and WT strains [145,146], illustrating the importance of this interaction to VV pathogenesis. Interestingly, the Vpu1 protein encoded by human immunodeficiency virus-1 also binds β-TrCP, suggesting that unrelated viruses have independently evolved mechanisms to antagonize β-TrCP function [147].

### 5.2. F14

*F14L* expression occurs early during VV infection, peaks between 4 and 8 h post-infection, and is not essential for viral replication [148]. A comparison of intranasal and intradermal infection with Δ*F14L*, knockout, and revertant strains showed that the F14 knockout is attenuated only in intradermally infected mice [148]. F14 mimics the “ΦΧΧΦΦ” motif found in the transactivation domain of p65 and exerts its inhibitory function on NF-κB in the nucleus by blocking the co-activation of NF-κB by CREB-binding protein (CBP/p300). Acetylation of the p65 subunit of NF-κB by CBP is required for the initiation of the transcription of pro-inflammatory genes such as CXCL10 and CCL2 [149], but is blocked by F14 binding [148]. Using cell lines expressing an inducible F14 construct, Albarnaz et al. showed that F14 drastically reduced the acetylation of p65. Notably, other viral proteins such as HIV-1 Tat, Adenovirus E1A, and HPV16 E6 also bind CBP [150,151,152], but F14 is the only known NF-κB inhibitor to mimic the transactivation domain of p65, and thus, represents a unique mechanism of viral immune evasion [148].

### 5.3. K1

Early work showed that K1 is a host-range factor because it recovers VV replication in a WR-strain *K1L*^−^/*C7L*^−^ mutant that is replication-incompetent in human cells [153,154]. However, it is not essential for the replication of WT VV strains expressing *C7L*, but has an impact on pathogenicity [92,153,155]. K1 is expressed early during infection like most of the VV NF-κB inhibitors, and inhibits NF-κB activation via two mechanisms. Insertion of WR *K1L* into the attenuated MVA strain (which normally activates NF-κB signaling) resulted in a blockage of IκBα degradation after the infection of RK13 rabbit cells [89]. A follow-up study found that IκBα degradation could still be observed in certain mammalian cells overexpressing *K1L* that were stimulated with TNFα, suggesting that the expression of K1 alone produces a cell-line-specific phenotype or is not sufficient on its own to block IκBα degradation [156]. This led to the discovery of a secondary mechanism by which K1 inhibits NF-κB, which is similar to F14. K1 inhibits the transcriptional activity of NF-κB by blocking p65 acetylation, consequently preventing the interaction of p65 with CBP in the nucleus [156].

### 5.4. A55

*A55R* was reported to be expressed both early and late during infection [157,158] and is non-essential for replication. A55 has been shown to influence VV virulence in an intradermal mouse model where an *A55R* deletion strain produces larger lesions compared to WT VV, suggesting that A55 reduces host immune response-mediated pathology during infection [158]. A55 is a member of the BTB/Kelch family of proteins, which regulate ubiquitin-mediated modification or the degradation of target proteins by acting as adaptors for the cullin-3 ubiquitin ligase complex [159]. Pallet et al. performed a luciferase reporter-based assay for NF-κB-dependent gene expression and showed that the Kelch domain, but not the BTB domain, was sufficient to inhibit NF-κB-dependent reporter activity when the two domains were expressed individually in cells that were stimulated by IL-1β or TRAF6 overexpression [160]. Subsequent experiments revealed that A55 interacts with importin a1 through its Kelch domain in order to prevent the translocation of NF-κB to the nucleus [160,161]. Given the many cellular proteins that interact with importin proteins to gain entry into the nucleus, it is likely that A55 may function to inhibit the nuclear import of additional host factors.

### 5.5. C4

The early protein C4 is non-essential and localizes to both the cytoplasm and nucleus during VV infection [162]. The effect of Δ*C4L* on virulence is dependent on the infection model. Intradermal mouse models reflect no change in lesion size between the WT and C4 knockout strains of VV. Comparing mice in an intranasal infection model with the same two viruses revealed the importance of Δ*C4L* for virulence, as indicated by reduced weight loss in the C4 knockout-infected group [162]. Using a reporter for NF-κB-dependent gene expression in tandem with TRAF2, TRAF6, or IKKβ overexpression, Ember et al. showed that C4 inhibits NF-κB downstream of IKKβ but prior to the nuclear translocation of active p65, although the exact host target of C4 remains unknown [162].

### 5.6. M2

M2 is expressed early during infection, and inactivation of the *M2L* gene does not affect VV replication in culture [163]. Gedey et al. showed that 293T cells infected with MVA (which lacks *M2L* [133]) and treated with U0126 and PD98059—which prevent the activation of extracellular signaling-regulated kinase1/2 (ERK1/2) [164]—decreased virus-induced NF-κB activity and suggested a role of ERK1/2 in VV-induced NF-κB activation [165]. 293T cells treated with phorbol myristate acetate and infected with MVA expressing WT M2L resulted in reduced phosphorylation of ERK2 and reduced p65 levels in the nucleus compared to cells infected with parental MVA [165]. M2 exhibits localization to the ER and possesses an N-terminal signal peptide sequence, a C-terminal ER-retention sequence, and N-glycosylation sites, the first two of which are important for its ER localization as well as its ability to inhibit NF-κB [166]. Hinthong et al. used radiolabeled oligonucleotides of NF-κB binding motifs to monitor the activation and migration of NF-κB throughout cells infected with WT MVA expressing functional M2L or M2L mutants lacking the signal peptide or ER localization sequence [166]. This study revealed the importance of both M2 sequences for inhibiting NF-κB since active NF-κB was only detected in the cells infected with parental MVA or MVA strains expressing either M2L mutants [165]. Exactly how the signal peptide and ER localization sequences of M2 contribute to the inhibition of ERK1/2-mediated NF-κB activation is still unclear.

## 6. Conclusions

In this review, we have examined the eighteen VV-encoded inhibitors of NF-κB characterized thus far and have categorized them based on their targeting of receptors mediating NF-κB activation, signaling intermediates, or the direct inhibition of NF-κB complexes (Table 1).

NF-κB plays a crucial role in regulating host innate immune responses against infection. This pathway is a critical target for viral evasion in general; however, the sheer number of non-redundant immunomodulators VV encodes highlights the importance of the host NF-κB signaling pathway in the response to poxvirus infection. As illustrated, VV has evolved a multifaceted approach to manipulate NF-κB-regulated gene expression to suppress host defenses (Figure 1).

Despite the focus of this review being on VV-encoded NF-κB inhibitors, it is important to highlight that homologous proteins of the aforementioned inhibitors exist in other members of the poxvirus family. For example, MC54L from molluscum contagiosum virus, C8L from cowpox, and vIL-18bp from ectromelia virus, all share significant sequence similarity with the VV C12L NF-κB inhibitor and have all been demonstrated to be soluble IL-18-binding proteins that block NF-κB activation [95]. Additionally, proteins related to VV inhibitors targeting NF-κB signaling intermediates, such as VV B13, can also be found in other poxviruses including CrmA in cowpox virus, and serp2 and S013L in leporipoxviruses myxoma virus and Shope fibroma virus, respectively [128,167,168,169,170].

In addition, some poxviruses have developed unique strategies to modulate NF-κB. For example, the gene product of ORFV002 from orf virus, a *parapoxvirus* infecting sheep and goats, has been shown to inhibit p300-mediated acetylation of the p65 NF-κB subunit by interacting with p65 and preventing p65–p300 association, which, in turn, inhibits NF-κB-dependent transcription [171,172]. Though there are two known VV inhibitors described to prevent p65 acetylation (K1 and F14), ORFV002 does so via an independent mechanism; it binds directly to p65 to inhibit acetylation, rather than inhibit upstream acetyltransferase activity [148,156,171]. Molluscum contagiosum virus also encodes a distinct NF-κB inhibitor, MC160, which has been shown to interact with heat shock protein (Hsp90), resulting in IκBα degradation and the inhibition of TNF-α-stimulated NF-κB induction [173]. Therefore, it is clear that poxvirus-mediated NF-κB inhibition is a widespread phenomenon extending beyond orthopoxviruses such as VV, and additional investigations of more poorly characterized poxviruses will undoubtedly reveal additional NF-κB antagonists.

Although many NF-κB inhibitors are non-essential for VV replication in cell culture, virtually all of them affect VV virulence in mouse models. In many cases, the inactivation of single VV NF-κB inhibitors leads to reduced viral replication and virulence, indicating a critical role for NF-κB-driven pro-inflammatory responses in viral clearance. However, in some infection models, the inactivation of VV NF-κB inhibitors (e.g., intradermal infection models with ΔB13R VV) leads to increased pathology due to exacerbated host inflammatory responses. This exemplifies the complex relationship between the NF-κB proinflammatory response and the pathogenesis associated with poxvirus infection, wherein NF-κB responses may be beneficial for viral clearance in certain contexts (e.g., routes of infection and tissue types) but pathogenic to the host in other cases. Furthermore, these observations suggest that, despite their overlapping functions in NF-κB inhibition, each of these inhibitors plays critical roles in modulating viral pathogenesis in vivo. Evidence of poxviruses evolving independent strategies to antagonize the NF-κB pathway underscores this as a critical aspect of poxvirus-mediated immune evasion.

Not only will the continued identification of VV-encoded immune evasion factors be critical for understanding how immune suppression contributes to poxvirus disease, but such inhibitors may, in turn, lead to a greater mechanistic understanding of the function and regulation of the host innate immune pathways these viral proteins target. 

## Figures and Tables

**Table 1:** VV-Encoded NF-κB Inhibitors.

**Inhibitors targeting receptors mediating NF-κB activation**					
**WR Gene**	**Copenhagen Gene**	**Expression**	**Localization**	**Mechanism of NF-κB Inhibition**	**Reference**
032	K1L	Early	Cytoplasmic	Limits dsRNA production to prevent PKR stimulation	[70,89,92]
034	K3L	Early	Cytoplasmic	eIF-2α mimic	[82,83,84]
059	E3L	Early/Late	Cytoplasmic	Inhibits PKR activation as an RNA-binding protein	[73,75,76,77]
196	B15R	Early	Extracellular	Inteleukin-1β-binding protein	[100,102,103]
013	C12L	Early/Late	Extracellular	Inteleukin-18-binding protein	[93,94,95]
**Inhibitors targeting NF-κB signaling intermediates**					
**WR Gene**	**Copenhagen Gene**	**Expression**	**Localization**	**Mechanism of NF-κB Inhibition**	**Reference**
028	N1L	Early/Late	Cytoplasmic	Inhibits IKK complex members, facilitating NF-κB activation	[126,127]
039	K7R	Early	Cytoplasmic	TRAF6 and IRAK2 interaction inhibiting NF-κB activation	[104,106]
056	F17R	Late	Cytoplasmic	mTOR dysregulation leading to cGAS degradation	[144]
172	A46R	Early/Late	Cytoplasmic	Targets TIR-domain-containing adaptor proteins (e.g., MyD88, Mal)	[107,109,112,113,114]
178	A52R	Early/Late	Cytoplasmic	TRAF6 and IRAK2 interaction inhibiting NF-κB activation; Targets host TIR domain-containing proteins (e.g., MyD88)	[109,117]
184	B2R	Early	Cytoplasmic	2′3′-cGAMP nuclease inhibiting cGAS-STING signaling	[141]
195	B13R	Early	Cytoplasmic	Blocks proteolytic activity of ICE/Caspase-1	[128,134]
196	B14R	Early	Cytoplasmic	Prevents IKKβ trans-auto-phosphorylation; Sterically hinders IKKβ-IKK complex formation	[121,122]
**Inhibitors directly targeting NF-κB complex activation/activity**					
**WR Gene**	**Copenhagen Gene**	**Expression**	**Localization**	**Mechanism of NF-κB Inhibition**	**Reference**
024	C4L	Early	Cytoplasmic	Prevents nuclear translocation of p65/RelA	[162]
031	M2L	Early	Cytoplasmic	ERK1/2 antagonist	[165,166]
032	K1L	Early	Cytoplasmic Nuclear	IκBα degradation inhibitor; Prevents acetylation of NF-κB subunit p65/RelA	[156]
053	F14L	Late	Nuclear	Inhibits acetylation of NF-κB subunit p65/RelA	[148]
175	A49R	Early/Late	Cytoplasmic	Interacts with β-TRCP to prevent ubiquitination of IκBα	[149]
180	A55R	Early	Cytoplasmic	Inhibits importin α-dependent nuclear translocation of NF-κB	[159,160,161]

## Data Availability

Not applicable.
